# Complete plastome sequence of *Monoon laui* (Merr.) B. Xue and R.M.K. Saunders, 2012: an endemic species in Hainan

**DOI:** 10.1080/23802359.2022.2116953

**Published:** 2022-09-07

**Authors:** Hao Xiu, Yirong Sun, Yunfan Quan, Tingting Fu, Qinghui Sun

**Affiliations:** a School of Tropical Medicine, Hainan Medical University, Haikou, 571199, China; b Key Laboratory of Tropical Translational Medicine of Ministry of Education, NHC Key Laboratory of Control of Tropical Diseases, School of Tropical Medicine, Hainan Medical University, Haikou, Hainan, 571199, China; c Modern Service Teaching and Research Office, Hainan Economic and Technical School, Haikou, Hainan, 571199, China

**Keywords:** *Monoon laui*, genome structure, plastome, phylogeny

## Abstract

*Monoon laui* (Merr.) B. Xue and R.M.K. Saunders 2012 is produced in Hainan province. The trunk is straight, the wood texture is straight, and the material is slightly soft, which is suitable for furniture and building materials. In our study, we report and characterize the complete plastome of *M. laui* The complete length of the plastome of *M. laui* possesses 161,181 bp, including a large single-copy (LSC) of 89,556 bp, small single-copy (SSC) of 18,977 bp, and two inverted repeats (IRs) of 26,313 bp. The overall G/C content in the plastome of *M. laui* is 39.13%. The plastome contains 257 genes, consisting of 130 protein-coding genes (16 of which are duplicated in the IR), 37 tRNA genes (seven of which are duplicated in the IR), and eight rRNA genes (5S rRNA, 4.5S rRNA, 16S rRNA, and 23S rRNA). Here, we explore the phylogenetic relationships and make contributions to the conservation genetics of the specie of *M. laui* using the complete plastome sequence.

## Introduction

*Monoon laui* (Merr.) B. Xue and R.M.K. Saunders 2012. belong to the family *Annonaceae* Juss. *M. laui* is a tree. The leaves are nearly leathery to leathery, oblong or oblong-elliptic, 8–20 cm long, 3.5–8 cm wide, acuminate at the top, broad and acute or rounded at the base, glabrous and shiny on both sides. It is produced in the low to mid-altitude forests of Hainan province. The trunk is straight, the bark is yellowish brown, and the flowers are pale yellow, which can be used as a greening and beautifying tree species for gardens and sidewalks. The wood is straight and slightly soft, suitable for furniture and building materials. At present, the systematic position of *M. laui* have not been reported. Hence, we used the complete plastid of *M. laui* (GenBank accession number: OL979152, this study) to explore the phylogenetic relationships, in order to facilitate the collection, preservation and systematic research of *M. laui* germplasm resources.

In this study, *M. laui* was sampled from Ledong county in Hainan province of China (108.79° E, 18.69° N). The leaves were deposited in silica gel. DNA was extracted from dried leaf tissue using a modified cetyltrimethylammonium bromide (CTAB) method, with chloroform: isoamyl alcohol separation and isopropanol precipitation at −20 °C. Its DNA was deposited in the Herbarium of the Institute of Tropical Agriculture and Forestry (code of herbarium: HUTB), Hainan University, Haikou, China.

The experiment procedure is as reported in Wang et al. (2021). Each individual is based on 6 Gb clean data, adapter sequences and low-quality reads with Q-value ≤20 were removed. Clean reads were assembled against the plastome of *Polyalthiopsis verrucipes* (MW018366.1) using MITObim v.1.8 (Hahn et al. 2013). The plastome was annotated using Geneious R8.0.2 (Biomatters Ltd., Auckland, New Zealand).

The result of our study indicates that the complete length of the plastome of *M. laui* possesses 161,181 bp with the typical quadripartite structure of angiosperms. This plastid contains an LSC region of 89,556 bp and an SSC region of 18,977 bp, two IRs of 26,313 bp. The plastome contains 257 genes, consisting of 130 protein-coding genes (sixteen of which are duplicated in the IR), 37 tRNA genes (seven of which are duplicated in the IR), and eight rRNA genes (5S rRNA, 4.5S rRNA, 16S rRNA, and 23S rRNA). The overall G/C content in the plastome of *M. laui* is 39.13%, while the corresponding value of the LSC, SSC, and IR regions were 37.7%, 34.3%, and 43.2%, respectively.

**Figure 1.**
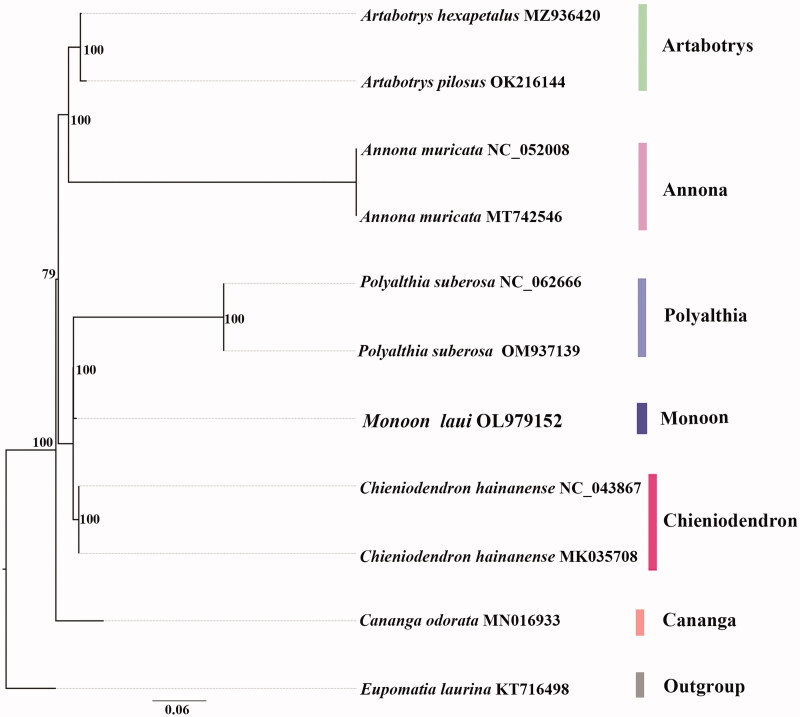
The best ML phylogeny recovered from 11 complete plastome sequences by RAxML. Accession numbers: *Monoon laui* (GenBank accession number, OL979152, this study), *Artabotrys hexapetalus*, MZ936420, *Artabotrys pilosus*, OK216144, *Annona muricata*, MT742546, *Annona muricata*, NC_052008, *Polyalthia suberosa*, OM937139, *Polyalthia suberosa*, NC_062666, *Chieniodendron hainanense*, NC_043867, *Chieniodendron hainanense*, MK035708, *Cananga odorata*, MN016933, *Eupomatia laurina*, KT716498.

We used IQ-TREE v.1.6.1 (Nguyen et al. 2015) to establish evolutionary relationships (Kalyaanamoorthy et al. 2017). We inferred the phylogeny of *M. laui* based on plastid alignments (Figure 1). The majority of nodes in the plastome ML trees were highly supported. The phylogenetic analysis indicates that *M. laui* is sister to the clade of *Polyalthia suberosa* (Roxb.) Thwaites having strong support (Figure 1).

Nowadays, the plastid sequence of *M. laui* has been gradually developed and tended to be perfect and phylogenetic studies of *M. laui* can be explored more sufficiently.

## Data Availability

The genome sequence data supporting the results of this study are publicly trustworthy in GenBank of NCBI (https://www.ncbi.nlm.nih.gov/)with registration number OL979152. Related BioProject, SRA, Biosample numbers are PRJNA748537, SRS12065959, and SAMN20703218, respectively. A specimen was deposited at Hainan University (https://ha.hainanu.edu.cn/home2020/,Qinghui Sun and hy0211068@hainmc.edu.cn) under the voucher number A209.
